# The Retinal Inner Plexiform Synaptic Layer Mirrors Grey Matter Thickness of Primary Visual Cortex with Increased Amyloid *β* Load in Early Alzheimer's Disease

**DOI:** 10.1155/2020/8826087

**Published:** 2020-09-21

**Authors:** Lília Jorge, Nádia Canário, Ricardo Martins, Beatriz Santiago, Isabel Santana, Hugo Quental, Francisco Ambrósio, Rui Bernardes, Miguel Castelo-Branco

**Affiliations:** ^1^Coimbra Institute for Biomedical Imaging and Translational Research (CIBIT), University of Coimbra, Coimbra, Portugal; ^2^Institute for Nuclear Sciences Applied to Health (ICNAS), University of Coimbra, Coimbra, Portugal; ^3^Faculty of Medicine, University of Coimbra, Coimbra, Portugal; ^4^CNC.IBILI Consortium, University of Coimbra, Coimbra, Portugal; ^5^Department of Neurology, Centro Hospitalar e Universitário de Coimbra (CHUC), Coimbra, Portugal; ^6^Coimbra Institute for Clinical and Biomedical Research (iCBR), Retinal Dysfunction & Neuroinflammation Lab, Coimbra, Portugal

## Abstract

The retina may serve as putative window into neuropathology of synaptic loss in Alzheimer's disease (AD). Here, we investigated synapse-rich layers versus layers composed by nuclei/cell bodies in an early stage of AD. In addition, we examined the associations between retinal changes and molecular and structural markers of cortical damage. We recruited 20 AD patients and 17 healthy controls (HC). Combining optical coherence tomography (OCT), magnetic resonance (MR), and positron emission tomography (PET) imaging, we measured retinal and primary visual cortex (V1) thicknesses, along with V1 amyloid *β* (A*β*) retention ([11C]-PiB PET tracer) and neuroinflammation ([11C]-PK11195 PET tracer). We found that V1 showed increased amyloid-binding potential, in the absence of neuroinflammation. Although thickness changes were still absent, we identified a positive association between the synapse-rich inner plexiform layer (IPL) and V1 in AD. This retinocortical interplay might reflect changes in synaptic function resulting from A*β* deposition, contributing to early visual loss.

## 1. Introduction

Alzheimer's disease is characterized by the presence of abnormal extracellular A*β* toxic deposits causing synaptic dysfunction, putative neuroinflammation due to microglia activation, and neuronal loss [1–6], which typically lead to progressive brain atrophy.

The impact of this pathology in the retina as well as other parts of the visual system in AD remains to be understood [7]. In fact, along with the numerous cognitive and neuropsychiatric manifestations, visual complains have often been reported in this condition [7–12] which include loss of contrast and color sensitivity [13–17], visual field loss [18, 19] and deficits in the perception of shape from motion [20–23].

Such visual impairments have been previously attributed to visual cortical damage, including V1 and visual associative areas [24–27]. However, more recently, it has been suggested that neural populations in the retina are also affected by similar pathophysiological mechanisms [28–30], even in the absence of visual cortical atrophy [12, 31].

As a part of the central nervous system (CNS), the retina shares multiple features with the brain, in terms of embryological development, anatomy, and function [32–35]. These similarities along with the visual changes observed in patients suffering from neurodegenerative diseases have justified the proposal that the retina may serve as a mirror to assess brain changes [10, 36–38]. In fact, the pathological hallmarks of AD, i.e., neuronal loss, A*β* plaques, and neurofibrillary tangles from the hyperphosphorylation of tau protein (pTau), have also been found in the retina [28, 39–43].

Nevertheless, despite all similarities between brain and retina, important biological differences also exist, which concerns the discovery of early disease biomarkers; some features of the retina are particularly prone to such investigation, in particular, the presence of synapse-rich layers. In the retina, neuron cell bodies are organized in specific layers, while the plexiform layers are exclusively dedicated to make the synaptic connection between the different kinds of neurons (for a review, see Hoon et al. [44]), and thus, synapse-rich and nuclei/cell body layers can be easily and independently assessed. For example, the GCL (ganglion cell layer) comprises ganglion cell bodies and displaced amacrine cells [45], while the IPL is densely packed with synapses connecting retinal ganglion cells, amacrine cells, and bipolar cells [44]. This allows testing the dominance of synaptic mechanism vs. cell loss and hence might let to detect subtle initial changes in dendritic integrity given the tenet that the loss of synapses may precede cell loss [46], being more prone to occur in patients with early AD.

In fact, evidence about neurotransmitter changes, structural alterations, and other biochemical markers favours the hypothesis that AD represents, at least in the early stages, a synaptopathy [47, 48]. Considerable evidence suggests that before massive neuronal cell death occurs, there is synaptic dysfunction originated by oligomeric assemblies of the A*β* protein in the hippocampus, one of the first brain structures affected by the pathological mechanisms of the disease [46]. Remarkably, this early loss of synaptic integrity was already demonstrated in the retina of an AD mouse model [49], as well as in ocular neurodegenerative diseases [50]. Therefore, retina might represent an excellent target to detect early neuropathological mechanisms in a faster, direct, and cost-effective way, using *in vivo* eye imaging techniques, than methods to assess the same mechanisms in the brain.

Existing work addressing structural changes in AD, using OCT, has focused primarily on both RNFL (retinal fiber layer) and GCL, and the controversy remains [8, 31, 51–57], whereby differences could appear only in the late stages of the disease [58].

In the present study, we aimed to take advantage of the existence of synaptic versus cell body-rich layers in the retina to investigate whether AD affects differently such layers, as compared to healthy controls, in relation to integrity of V1—the visual area that primarily receives inputs from the retina. To that end, we measured in V1 A*β* levels through [11C]-Pittsburgh Compound B (PiB), neuroinflammation using [11C]-PK11195 radiotracer, and neuronal loss by means of cortical thickness analysis. Regarding the retina, we studied the structural integrity of 4 layers, 2 composed by synapses—IPL and OPL (outer plexiform layer)—and 2 composed by cell bodies—GCL (ganglion cell layer) and INL (inner nuclear layer), the layers of the inner retina closer to the brain.

Taking into account the substantial evidence suggesting that synaptic loss is one of the earliest pathological changes in AD, we hypothesize that synapse-rich layers would better reflect cortical status, considering the early stage of our AD sample.

To the best of our knowledge, the present work is the first to investigate an explicit disease-related morphometric and molecular association between V1 and retinal integrity in AD. Considering the uncertainty of the relative role of visual cortex or retina in the visual deficits observed in this population, it is crucial to study both structures and their potential relationships to common disease mechanisms.

## 2. Methods

### 2.1. Participants

A total of 41 subjects were recruited in the present study. We included 20 AD patients with a probable diagnosis supported by biological biomarkers (CSF and PET-PiB) and in mild stages of the disease, according to the Clinical Dementia Rating (CDR = 1). Patients were recruited at the Neurology Department of Coimbra University Hospital. The diagnosis criteria of AD were based on the Diagnostic and Statistical Manual of Mental Disorders–fourth edition (DSM-IVTR) [59] and the National Institute of Neurological and Communicative Disorders and Stroke-Alzheimer's Disease and Related Disorders (NINCDS-ADRDA) [60]. A comprehensive neuropsychological evaluation battery was administered, including (1) cognitive instruments as the Mini-Mental State Examination (MMSE) with Portuguese normative data [61, 62], the Montreal Cognitive Assessment (MoCA) [63, 64], and a comprehensive neuropsychological battery with normative data for the Portuguese population (BLAD) [65] exploring memory and other cognitive domains.

We considered that patients had to be in a stable condition, without acute significant events or recent/undergoing changes in medication; we defined this as exclusion criteria ophthalmological comorbidities or neurological/psychiatric conditions other than AD or CT or MRI demonstration of significant vascular burden (large cortico-subcortical infarct; extensive subcortical white matter lesions superior to 25%; uni- or bilateral thalamic lacune; lacune in head of caudate nucleus; more than 2 lacunes) (8) [60].

For the present study, we selected AD patients with a probable diagnosis supported by biological biomarkers (cerebrospinal fluid (CSF) or PiB-PET). The cut-off values used in our laboratory and applied in the present study were 580 pg/mL for A*β*_1–42_, 0.068 for A*β*_42_/A*β*_40_, 250 pg/mL for tau, and 37 pg/mL for pTau181 (Table 1).

The control group was composed of 21 individuals matched for age, education, and sex, from the community, with no history of CNS, neurodevelopmental, or mental disorders. This group was also submitted to cognitive assessment and showed no significant memory complaints (Subjective Memory Complaints Questionnaire-SMC ≤ 3) [66, 67], had a normal general cognitive status tested by the MoCA (mean ± standard deviation (SD), 24.88 ± 4.24), had preserved daily living activities (Lawton and Brody scale—for female = 8; for male = 5) [68, 69], and no evidence of moderate or severe depressive symptoms (30-item Geriatric Depressive Scale – GDS-30, mean ± SD6.41 ± 6.20) [70, 71].

All subjects underwent PET imaging with [11C]-PiB and [11C]-PK11195 radiotracers in two different visits with a maximum interval of 1 month. PiB positivity was determined by an experienced nuclear medicine physician, who considered simultaneously a visual regional SUVR (standard uptake value ratio) analysis and the output of a homemade support vector machine classifier. The visual analysis of these images was regional based with emphasis to frontal cortex, parietal/precuneus cortex, temporal cortex, anterior and posterior cingulate cortex, basal ganglia, and occipital cortex.

Four subjects of the control group we found with PiB positive, an increasingly frequent finding, which led to their exclusion, although the cognitive tests performed were within normal ranges.

None of the participants had history of ocular diseases, and all were submitted to a comprehensive ophthalmological examination to guarantee the absence of visual complications, which comprised visual acuity assessment with Snellen chart, ocular tension, slit lamp biomicroscopy, and OCT imaging. We involved only subjects with normal or corrected to normal vision (visual acuity ≥ 8/10), with a refractive error between ±5 diopters, and without significant alterations of the optic disc or macula. Additionally, we considered exclusion criteria family history of glaucoma, or any other hereditary eye disease and diabetes or other systemic diseases that could affect the eye.

The study was approved by the Ethics Committee of the Faculty of Medicine, University of Coimbra. All subjects participated voluntarily and gave their informed written consent for the study, following the tenets of the Declaration of Helsinki, after clarification of the nature and possible implications of the study.

### 2.2. Retinal Imaging

Imaging data from the retina of both eyes were obtained by optical coherence tomography through the Cirrus HD-OCT system (Carl Zeiss Meditec, Inc., Dublin, CA, USA) with the macular cube 512 × 128 protocol, by one experienced technician (HQ). This protocol acquires data through a 6 mm square grid centered on the fovea by acquiring a series of 128 horizontal B-scan lines, each composed of 512 A-scans, with an axial resolution of 5 *μ*m.

### 2.3. MR Imaging

Brain structural data were acquired using a whole-brain approach, with a phased array 12-channel birdcage head coil, in a Siemens Magnetom TIM Trio 3 Tesla scanner (Siemens, Munich, Germany). For each participant, one high-resolution T1-weighted three-dimensional Magnetization Prepared Rapid Acquisition Gradient Echo (MPRAGE) was acquired, with the following acquisition parameters: 1.0 × 1.0 × 1.0 mm^3^ voxel resolution, repetition time (TR) 2530 ms, echo time (TE) 3.42 ms, and field of view (FOV) 256 × 256 mm. The anatomical sequence comprised 176 slices, a flip angle of 7°, and an inversion time of 1100 ms.

### 2.4. PET Imaging

The [11C]-PiB PET and [11C]-PK11195 PET acquisitions were performed using a Philips Gemini GXL PET/CT scanner (Philips Medical Systems, Best, the Netherlands). Both acquisitions consisted of dynamic 3-dimensional PET scan of the entire brain (90 slices, 2 mm slice sampling) and a low-dose brain computed tomography (CT) scan, for attenuation correction. The dynamic [11C]-PiB PET image comprised 24 frames (total duration of 90 minutes: 37 frames: 4 × 15 s + 8 × 30s + 9 × 60s + 2 × 180 s + 14 × 300 s) and the dynamic [11C]-PK11195 image of 22 frames (total duration of 60 minutes: 4 × 30 s + 4 × 60 s + 4 × 120 s + 4 × 240 s + 6 × 300 s). The [11C]-PiB PET or [11C]-PK11195 PET image acquisition sessions started immediately after the intravenous bolus injection of approximately 555 MBq of [11C]-PiB or 370 MBq of [11C]-PK11195. To minimize head movement, the patients' head was restrained with a soft elastic tape. The PET images were reconstructed to a 128 × 128 × 90 matrix, with 2 mm isotropic voxel dimension, using the LOR RAMLA algorithm (Philips PET/CT Gemini GXL) with attenuation and scatter correction.

### 2.5. Cortical Thickness Assessment

Brain imaging processing was conducted in SPM12 software (Wellcome Trust Centre for Neuroimaging, Institute of Neurology, UCL, London, UK) throughout its computational anatomy toolbox (CAT12) (http://dbm.neuro.uni-jena.de/cat/), which allows fully automatic cortex segmentation and cortical thickness measurements.

The anatomic images were firstly reoriented into the AC-PC plane and then automatically corrected in order to diminish the intensity variations caused by the magnetic field and RF-field inhomogeneities. Thereon, automatic cortex segmentation in volume space of white matter-grey matter (WM-GM) and grey matter-cerebrospinal fluid (GM-CSF) boundaries was performed relying on prior probability tissue maps, assigning to each voxel a value representing the proportion of the corresponding tissue type [72].

To the spatial normalization, the subject's brains were aligned to a standard MNI template, resorting to the high-dimensional registration DARTEL algorithm in SPM [73]. At the end of the automatic segmentation procedure, a smoothing on the normalized GM maps was applied by a 15 mm isotropic Gaussian kernel; the datasets were then submitted to well-suited fully automated thickness measurements based on the projection-based thickness method, as described by [74]. This method creates both a correct cortical thickness map and the central cortical surface in one step. The subsequent analyses were performed in surface space, which allows to reparametrize the surface mesh into a common coordinate system throughout a spherical mapping, improving the correspondence between individual subject's areas.

**Figure 1:**
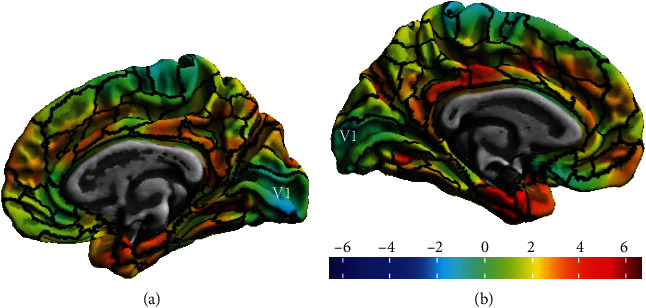
Resulting cortical thickness map between HC and AD groups, with the representation of the V1 area: (a) right hemisphere, (b) left hemisphere.

Moreover, the CAT12 toolbox allows the estimation of ROI-based mean thickness values relying on internal surface maps [75]. We used the ROI-based values provided by the Human Connectome Project (HCP's) multimodal parcellation [76] surface map, which comprises anatomically delineated V1 (see Figure 1). ROI-based thickness measures were extracted for each subject and then imported by SPSS for further statistical analysis.

### 2.6. Retinal Thickness Assessment

**Figure 2:**
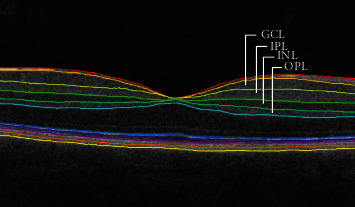
Macular image resulting from the segmentation, identifying the layers analyzed in this study. GCL: ganglion cell layer; IPL: inner plexiform layer; INL: inner nuclear layer; OPL: outer plexiform layer.

The OCT datasets underwent an automatic segmentation routine using the Iowa Reference Algorithms software (version 4.0.0, Retinal Image Analysis Lab, Iowa Institute for Biomedical Imaging, Iowa City, IA, USA) [77–79], providing the segmentation of retinal nerve fiber layer (RNFL), ganglion cell layer (GCL), inner plexiform layer (IPL), inner nuclear layer (INL), outer plexiform layer (OPL), outer nuclear layer (ONL), inner segment/outer segment junction (IS/OS), outer segment (OS), outer segment photoreceptor/RPE complex (OPR), and retinal pigment epithelium (RPE). In this study, we focused on the individual layers from CGL to OPL (see Figure 2).

Each of the 128 B-scans and all the 5 surface layers were visually inspected in order to check the quality of the segmentation. Manual corrections were performed just in case of an evident algorithm failure. Subsequently, the distance (in voxels) between the respective delimiting surfaces was taken as the thickness of each layer, multiplied by the voxel size in that direction. These measurements were provided by the software based on the number of voxels and imaging depth.

We focused in central vision due to the central magnification present in V1, and given the evidence of a higher involvement of the central vision in the disease [13, 80, 81]. The thickness of each macular layer was computed as the average from a whole area of a 3 mm diameter circular map centered on the foveal pit, corresponding to inner macular ring of the standard EDTRS chart and to an eccentricity of 9.4°.

### 2.7. PET Imaging Preprocessing and Quantitative Analysis

A sum image obtained using all the frames of the dynamic PET was used to estimate a rigid transformation between the [11C]-PiB PET image space or [11C]-PK11195 PET image space and the T1 anatomical MRI space of each participant, using the 3D-Slicer software (version 4.8.1, BRAINS registration tool, http://www.slicer.org).

The individual MRI scans were spatially normalized to an MNI template using the DARTEL algorithm in SPM12.

The voxel-level quantitative analysis of [11C]-PiB PET images and [11C]-PK11195 PET images was implemented in the MNI space using in-house made software. The individual [11C]-PiB standard uptake value ratio (SUVR) map was computed by summing voxel-level signal from 40 to 70 min postinjection, and dividing by the mean signal from the individual's reference region, the cerebellar grey matter (essentially the cerebellum without the cerebellar peduncles) [82–84]. The individual [11C]-PK11195 binding potential (BP_ND_) maps were generated using the MRTM2 (Multilinear Reference Tissue Model 2) [85]. The reference region was determined by the algorithm SVCA4 (Supervised Cluster Analysis with 4 classes: grey matter without specific binding, white matter, blood, grey matter with specific binding) [86], which selected a group of grey matter voxels showing a time-activity curve representing the kinetic activity of normal grey matter without [11C]-PK11195 specific binding.

To extract [11C]-PiB uptake and [11C]-PK11195 BP_ND_ in V1, we resorted also to the CAT12 toolbox, following a similar procedure to that one used to measure the V1 thickness values. For each participant, we first mapped each PET volume dataset in native space to the respective individual surfaces, created during the cortical thickness measurement procedure, and then, we extracted the ROI-based values using HCP's multimodal parcellation [76] surface map, which comprises anatomically delineated V1 (Figure 1), the same used for the cortical thickness measurements. Average values of [11C]-PiB uptake and [11C]-PK1119 BP_ND_ in V1 were extracted from both hemispheres to each subject.

## 3. Statistical Analysis

Twenty mild-stage AD amyloid-positive patients and 17 amyloid-negative HC were considered for both retinal and cortical analyses. After the data normality was assessed using the Shapiro-Wilk test, *T*-test for unpaired samples or its nonparametric version and Mann-Whitney *U* test were used for between-group comparisons of the demographic data.

Average values of [11C]-PiB uptake, [11C]-PK11195 BP_ND_, and thickness in V1 were estimated for each participant across both hemispheres. Concerning the retina, a mean thickness value of both eyes was calculated for each analyzed macular layer. Independent-samples *t*-tests were computed for between-group comparisons of retinal layers thickness and V1-measured biomarkers, with exception of OPL thickness for which the Mann-Whitney *U* test was used, given the nonnormality of the data. Thereon, partial correlation analyses between the retina thickness and V1-driven measures (thickness, A*β* load and microglia activation) were computed for each group to identify possible associations between cortex AD-related biomarkers and retina integrity, controlling for age.

Data analysis was performed with IBM SPSS Statistics (version 22.0), and GraphPad Prism (version 6.0) was used for graphs and for slope analysis. The tests were performed two-tailed, and a threshold of *p* < 0.05 was used for statistical significance.

## 4. Results

Concerning the demographic data, no difference in age, sex, or education was found between groups, whereas results from cognitive assessment (MoCA) were significantly different between groups, as expected (Table 2). The BLAD battery confirmed that mnestic deficits were beyond cut-off in virtually all patients (95%), as compared to executive function (70%), language (40%), constructive (25%), and calculation deficits (20%).

**Figure 3:**
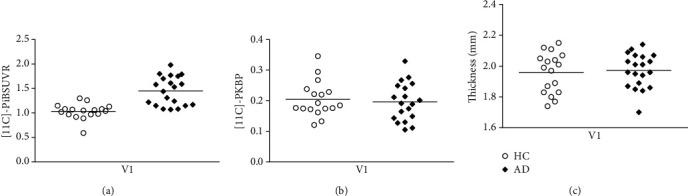
V1 measurements in 20 AD patients compared to 17 HC. (a) [11C]-PiB SUVR; (b) [11C]-PK1195 BP_ND_; (c) V1 thickness. There is a significant difference in the mean of [11C]-PiB SUVR (a) between groups, but not in [11C]-PK1195 BP_ND_ (b) or in V1 thickness (c).

Mean ± SD values of all variables included in the analysis are depicted in Table 3. Independent-samples *t*-tests did reveal significant differences between groups regarding the V1 [11C]-PiB uptake (see Figure 3). This shows that amyloid load is already affecting V1, in spite of overall preserved thickness. No evidence for significant neuroinflammation was found as assessed by [11C]-PK11195 BP_ND_.

The evidence for significant A*β* load in the absence of significant differences in visual pathways thickness assessment is consistent with the notion that our AD patients are at an early stage.

**Figure 4:**
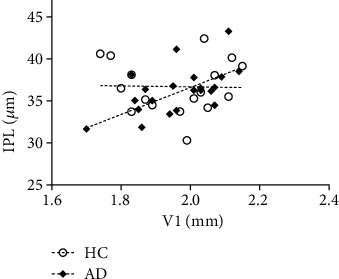
Scatterplot graph for the V1 thickness and IPL thickness to HC and AD.

We then asked whether partial (corrected) correlation analysis, performed to study the relationship between the retina and V1, could identify distinct patterns in patients and controls. Results showed a positive correlation between the IPL and V1 thicknesses in AD group (IPL: *r* = 0.604, *p* < 0.006, Bonferroni corrected for multiple comparisons) (Figure 4), whereas no significant associations were found with the other layers (GCL: *r* = 0.130, *p* < 0.595; INL: *r* = 0.450, *p* < 0.053; OPL: *r* = −0.295, *p* < 0.221). Considering the HC group, no significant correlation could be found between the V1 and the thickness of retinal layers. In spite of the significant structural-structural correlations, no significant structural-molecular correlations were found between the retina and V1.

Since we found evidence that V1 is already accumulating A*β*, which also target synapses early on, this positive correlation might suggest initial synaptic AD-related changes in the IPL, which is a specifically synapse-rich region in the retina accumulating amyloid both in animals [87] and humans [40]. Such association makes us suggest that this layer might serve as a biomarker for AD since the assessment of AD-related synaptic changes in the retina is faster, direct, and cost-effective way, using in vivo eye imaging techniques, than methods to assess the same mechanisms in the brain.

## 5. Discussion

In the present study, we were able to test whether in an early stage of the AD, the neuropathological mechanisms of the disease differently affects the integrity of retinal layers with distinct dominance of either synapses or cell bodies/nuclei between patients and healthy controls, matched for age, education, and sex. We sought for structure-structure associations (by computing partial correlations) at the level of the retina and cortex, while assessing putative biomarkers of the disease in V1—cortical thickness, neuroinflammation, and A*β* load.

Interestingly, AD patients presented higher levels of A*β* in V1 compared to controls, but no evidence of neuroinflammation. Together with the relatively preserved structural integrity, our results further suggest a relatively early disease stage. Importantly, a positive correlation between V1 thickness and the central IPL was specifically found in the AD group, suggesting a relationship between the eye and brain in the presence of A*β* load even when neuronal loss is not evident.

This striking association between a layer composed by synapses (IPL), closer to the cortex, and V1, suggests initial synaptic changes in the disease. This is an interesting finding considering previous animal studies referring IPL as a potential biomarker for the disease [49].

Taking into account current findings in AD research, namely, the evidence revealing that abnormal deposition of A*β* leads initially to synaptic dysfunction, evolving posteriorly to neuroinflammation and neuronal loss [46, 47], we suggested that in an early stage of the disease, V1 starts suffering from abnormal accumulation of A*β*, prior to detectable neuroinflammation or neuronal loss, which explains a structural association between this region and a macular layer rich in synapses. This pattern of early loss is consistent with cumulative reports suggesting that primary visual cortex is one of the later regions affected by the pathophysiological mechanisms of the disease, namely, in terms of neurofibrillary tangles (NFT), neuritic plaques (NP), and lesion load [88–90].

Furthermore, since we studied two synapse layers (IPL and OPL), the fact that an association was limited to IPL could be attributed to the fact that this layer is the one close to the brain, possibly suffering at first, at least partially, effects from the retrograde V1 deterioration.

Although visual deficits are one of the earliest manifestations of AD [11], there are few studies addressing specifically V1. Nevertheless, one functional study found changes in the visual field map organization and population receptive field measurements of V1 in two AD patients. In addition, one of the subjects had no changes in V1 surface area, while the other showed a reduced surface area in the most central visual field of V1, whereas the peripheral area showed the opposite pattern, when compared to controls [81].

Regarding the retina, a wide variety of studies has been conducted to assess its microstructural changes but results are still contradictory [8, 30, 53, 58, 91–93]. In particular, a study suggested that both RNFL and GCL were reduced in the AD sample, whereas the external layers did not show significant differences [94]. Moreover, a GC-IPL shrinkage was found across all macular quadrants in an AD as well as in a mild cognitive impairment (MCI) group [95]. In turn, another study found no difference in GC-IPL thickness in AD compared to the control group [96], similarly to our results. Likewise, a recent study in an early-onset AD patient sample showed no significant differences in the thickness of none of the 7 layers segmented, including GCL, IPL, INL, and OPL in this group when comparing to controls [54].

Accordingly, and in contrast with our work, the majority of the studies have assessed patients in mild-to-moderate or moderate-to-severe stage of the disease and with no information of A*β* deposition in AD and control groups. Thus, since AD is defined by progressive neurodegeneration, it would be expected a higher decay of structural integrity in the later stages, mainly in layers composed by neuron cell bodies, resulting from the neuronal loss, in contrast with synapse loss predominant in the initial stages.

Recently, some studies have investigated whether neuronal changes in the retina are associated with other forms of structural changes, such as connectivity assessed with MRI. Particularly, in a work of our group was found a significant relationship between INL and white matter integrity (axial diffusivity) of some cortical regions, including tracts associated with the visual system (optic radiation, splenium of the corpus callosum) [97], providing independent evidence for retinocortical associations. Other study searched for associations between CG-IPL and cortical integrity in three different groups. Results demonstrated that GC-IPL shrinkage was related with smaller GM volumes and WM microstructure in healthy participants in some regions, namely, in occipital cortex and cerebellum. In turn, no association was found in the MCI or AD groups [96]. A recent study from our group also found retinocortical associations in healthy aging [98].

Finally, by means of MRI visual rating scores for cortical atrophy, a correlation in both AD and control groups between total macular thickness and parietal atrophy was reported [54]. This issue was also addressed in a sample largely composed of nondemented participants with cognitive decline, where the association of peripapillary RNFL and macular GCL-IPL thickness with cerebral GM volume was examined. An association between the reduction of occipital and temporal lobes GM volumes with GC-IPL and peripapillary RNFL thickness was found. Moreover, because dendritic atrophy might take place before retinal ganglion cell loss, it was suggested that GC-IPL could be more sensitive to the neurodegenerative process [99].

Based on the notion that synapses are an initial target of the pathological mechanism of the disease [46], an animal study has tested whether the GCL dendritic integrity might provide a marker for cerebral damage in a mouse model of amyloid pathology. By evaluating animals carrying significant cognitive deficits resulting from the cortical deposition of A*β* plaques, the authors verified that although GCL significant loss was absent, there was already significant GCL dendritic atrophy in Tg2576 mice compared to controls. They proposed that dendritic changes precede cell loss and are, therefore, likely to occur in patients with early AD. Furthermore, they raised the hypothesis that in case of a resembling GCL degenerative pattern occurs in the human retinas, the IPL would be a useful biomarker for the early detection of AD-related neurodegeneration [49].

This question was directly addressed in the present work. We report here a striking positive correlation between IPL—the layer comprising synaptic connections between dendrites of ganglion cells and other retinal neurons—and V1 in the AD group that was not present in the control group. Hence, our finding might suggest a possible subtle synaptic/dendritic failure of the retinal CG cells that might be associated with primary visual cortex integrity. In fact, considerable evidence suggests that before a massive neuronal cell death, there is synaptic dysfunction originated by oligomeric assemblies of the A*β* protein in some cortical regions [46, 48]. Thus, in case of the mechanisms underlying the AD can be transposed to the retina, the IPL would be one of the layers more prone to suffer changes in the initial stages of the disease.

Consequently, we propose that prior to tissue loss in the retina, changes in synaptic function and dendritic morphology might occur resulting from A*β* deposition, which could contribute to the earlier decline of the visual abilities involved so remarkably in the disease. Nevertheless, along with further studies better suited to directly examine the retinal synaptic function, it is still important to define the nature of the biological mechanisms underlying visual deficits in AD.

In the present study, we were able to study in detail the visual system in Alzheimer's disease by comparing V1 measurements of AD key features and by investigating differences in the retina thickness of layers with distinct dominance of synapses/neurons cell bodies.

Our results aid in the understanding of visual processing pathway changes in AD patients and might open the door to future work addressing the IPL, using more sensitive techniques to detect synaptic changes in the early stages or prior to manifestation of AD symptoms, so that it could potentially be used as a potential biomarker for diagnosis and monitoring of the disease.

## Figures and Tables

**Table 1:** AD patients' CSF biomarker levels.

Variable	A*β*_1–42_ (*n* = 17)	A*β*_42_/A*β*_40_ (*n* = 14)	Tau (*n* = 17)	pTau (*n* = 17)	Tau/A*β*_42_ (*n* = 17)	A*β*_42_/pTau (*n* = 17)
Mean	510.94	0.057	445.64	63.15	0.98	9.23
SD	215	0.021	246.80	24.36	0.52	6.28

A*β*: amyloid beta; pTau: phosphorylated tau; SD: standard deviation.

**Table 2:** Demographic features of all participants. Age and education show no significant differences between groups, but a significant difference was found in MoCA.

Variable	AD group (*n* = 20) (mean ± SD)	HC group (*n* = 17) (mean ± SD)	Excluded HC (*n* = 4) (mean ± SD)	*p* value^∗^
Age (years)	65.294 (6.459)	66.250 (6.866)	68.5 (6.191)	0.667
Education (years)	11.412 (5.063)	9.300 (5.930)	9 (3.830)	0.239
Female/male ratio	10/10	7/10	2/2	—
MMSE	23.1 (2.97)		—	—
MoCA	14.35 (4.021)	24.88 (4.208)	23 (4.397)	<0.001
CDR	1	—	—	—

MMSE: Mini-Mental State Examination; MoCA: Montreal Cognitive Assessment; CDR: Clinical Dementia Rating; SD: standard deviation; ^∗^statistical tests performed between AD an HC groups.

**Table 3:** Mean V1 thickness (mean ± SD), V1 [11C]-PiB SUVR (mean ± SD), V1 [11C]-PK11195 BP_ND_ (mean ± SD), and retinal layers thicknesses (mean ± SD) per group. V1 [11C]-PiB uptake showed stark differences in A*β* load.

Variable	AD group (*N* = 20)	HC group (*N* = 17)	*p* value
V1 (mm)	1.974 (0.114)	1.957 (0.133)	0.719
V1 [11C]-PiB SUVR	1.4350 (0.290)	1.033 (0.156)	<0.0001^∗^
V1 [11C]- PK11195 BP_ND_	0.196 (0.060)	0.205 (0.058)	0.603
GCL (*μ*m)	41.19 (3.59)	42.067 (3.99)	0.488
IPL (*μ*m)	36.70 (3.16)	36.15 (2.80)	0.579
INL (*μ*m)	36.82 (1.99)	36.50 (2.10)	0.646
OPL (*μ*m)	30.42 (3.97)	28.41 (2.17)	0.104

V1: primary visual cortex; [11C]-PiB SUVR: A*β* PET radiotracer; [11C]-PK11195 BP_ND_: neuroinflammation PET radiotracer binding potential; GCL: ganglion cell layer; IPL: inner plexiform layer; INL: inner nuclear layer; OPL: outer plexiform layer; ^∗^Bonferroni corrected for multiple comparisons.

## Data Availability

We will be very glad to share the data of the present manuscript upon request to the corresponding author.
